# Deep learning–based temporal MR image reconstruction for accelerated interventional imaging during in-bore biopsies

**DOI:** 10.1117/1.JMI.12.3.035001

**Published:** 2025-06-03

**Authors:** Constant R. Noordman, Steffan J. W. Borgers, Martijn F. Boomsma, Thomas C. Kwee, Marloes M. G. van der Lees, Christiaan G. Overduin, Maarten de Rooij, Derya Yakar, Jurgen J. Fütterer, Henkjan J. Huisman

**Affiliations:** a Radboud University Medical Center, Department of Medical Imaging, Nijmegen, The Netherlands; b Isala, Department of Radiology, Zwolle, The Netherlands; c University Medical Center Groningen, Department of Radiology, Groningen, The Netherlands; d Netherlands Cancer Institute, Department of Radiology, Amsterdam, The Netherlands; e University of Twente, Technical Medical Centre, Robotics and Mechatronics Group, Enschede, The Netherlands

**Keywords:** artificial intelligence, deep learning, image processing (computer-assisted), magnetic resonance imaging, interventional radiology, neural networks (computer)

## Abstract

**Purpose:**

Interventional MR imaging struggles with speed and efficiency. We aim to accelerate transrectal in-bore MR-guided biopsies for prostate cancer through undersampled image reconstruction and instrument localization by image segmentation.

**Approach:**

In this single-center retrospective study, we used 8464 MR 2D multislice scans from 1289 patients undergoing a prostate biopsy to train and test a deep learning–based spatiotemporal MR image reconstruction model and a nnU-Net segmentation model. The dataset was synthetically undersampled using various undersampling rates (R=8, 16, 25, 32). An annotated, unseen subset of these data was used to compare our model with a nontemporal model and readers in a reader study involving seven radiologists from three centers based in the Netherlands. We assessed a maximum noninferior undersampling rate using instrument prediction success rate and instrument tip position (ITP) error.

**Results:**

The maximum noninferior undersampling rate is 16-times for the temporal model (ITP error: 2.28 mm, 95% CI: 1.68 to 3.31, mean difference from reference standard: 0.63 mm, P=.09), whereas a nontemporal model could not produce noninferior image reconstructions comparable to our reference standard. Furthermore, the nontemporal model (ITP error: 6.27 mm, 95% CI: 3.90 to 9.07) and readers (ITP error: 6.87 mm, 95% CI: 6.38 to 7.40) had low instrument prediction success rates (46% and 60%, respectively) compared with the temporal model’s 95%.

**Conclusion:**

Deep learning–based spatiotemporal MR image reconstruction can improve time-critical intervention tasks such as instrument tracking. We found 16 times undersampling as the maximum noninferior acceleration where image quality is preserved, ITP error is minimized, and the instrument prediction success rate is maximized.

## 1 Introduction

Interventional magnetic resonance imaging (iMRI) faces challenges related to speed and operational efficiency. Incorporating fast instrument-tracking technologies could significantly enhance the efficiency and efficacy of MR-guided intervention.^1^^,^^2^ This study focuses on speeding up transrectal in-bore MR-guided biopsy (MRGB) for prostate cancer, a common MR-targeted biopsy technique following diagnostic multiparametric MRI of the prostate.^3^

MRGB is rated less favorable in terms of time efficiency and ease of use compared with alternative techniques such as MRI-ultrasound fusion biopsy.^3^^–^^5^ However, recent retrospective studies highlight that MRGB excels in accurately identifying clinically significant cancers, yielding a higher rate of true positive biopsies.^6^^,^^7^ Enhancing the speed of MR acquisition during MRGB could be a crucial step toward achieving real-time instrument tracking in iMRI procedures.

Accelerating acquisitions are largely done by undersampling the k-space (frequency domain) data, which introduces aliasing artifacts in the resulting image. Although traditional methods have found some success in speeding up acquisition by undersampling data beyond the Nyquist sampling limit, they are limited to handling only moderate levels of undersampling.^8^ Deep learning–based image reconstruction models have shown promise to handle significantly increased levels of undersampling as these models effectively learn and leverage information from past datasets.^9^ However, these results do not necessarily translate to improved real-time tracking of instruments for iMRI. To address this knowledge gap, we introduce a novel approach that simulates temporal dynamics in prostate MRGB. Temporal refers to models sharing information across multiple time data frames rather than treating each image independently; in our setup, this is simulated by treating adjacent slices as sequential timepoints. This enables the use of temporal deep learning models in a domain where true temporal sequences are not routinely available.

Dynamic MRI monitors dynamic processes such as cardiac and instrument motion by acquiring both temporal and spatial data. It has real-time sequences with instrument tracking aided by active or passive instrument tracking, but its operational complexity restricts its acceptance in clinical practice.^10^^–^^12^ Recent research in cardiac cine MRI has shown that temporal deep learning reconstruction models can exploit spatiotemporal redundancies and significantly improve reconstruction quality.^13^

Temporal deep learning reconstruction methods applied to highly undersampled iMRI may make real-time instrument tracking feasible. This work explores the benefits of spatiotemporal exploitation in synthetically undersampled MRGB images and how it may improve results for the downstream task of tracking the needle guide instrument. We hypothesize that the model’s ability to use information over time significantly enhances the quality of reconstructed images, allowing for accurately predicting the needle guide instrument tip position (ITP) at higher undersampling rates. Specifically, we predict that our model has a higher maximum noninferior undersampling rate compared with a conventional nontemporal model. In addition, we compare ITP between AI and radiologists in a multicenter reader study to substantiate our results and create a human reference standard.

## 2 Materials and Methods

### 2.1 Dataset

The study was approved by the institutional review board at our center (identifier: CMO 2016-3045, Project 20011). Informed consent was exempted due to the retrospective scientific use of deidentified patient data. All patient data from January 2014 through December 2022 were consecutively included. For patient cohort selection details, see Sec. S1 in the Supplementary Material. The image-space MRGB dataset comprised 8464 intraprocedural, fully sampled, balanced steady-state free-precession DICOM scans from 1289 male patients, aged between 44 and 87 (median age 68), who were suspected of having prostate cancer. These patients underwent transrectal MRGB using a transrectal needle guide instrument, remotely controlled by a robotic prostate biopsy system.^14^ The scans, which image the pelvis, prostate, and instrument, are typically used for navigating the needle guide instrument and for the final assessment of biopsy quality. The data were acquired on a 3T scanner (Siemens Healthineers, Erlangen, Germany) with the following imaging parameters: 2.28 ms echo time, 4.56 ms repetition time, 70 deg flip angle, at a resolution of 256×256×5  voxels, with a 1.094×1.094×3  mm voxel size, and a total scan time of 7.3 s.

**Fig. 1:**
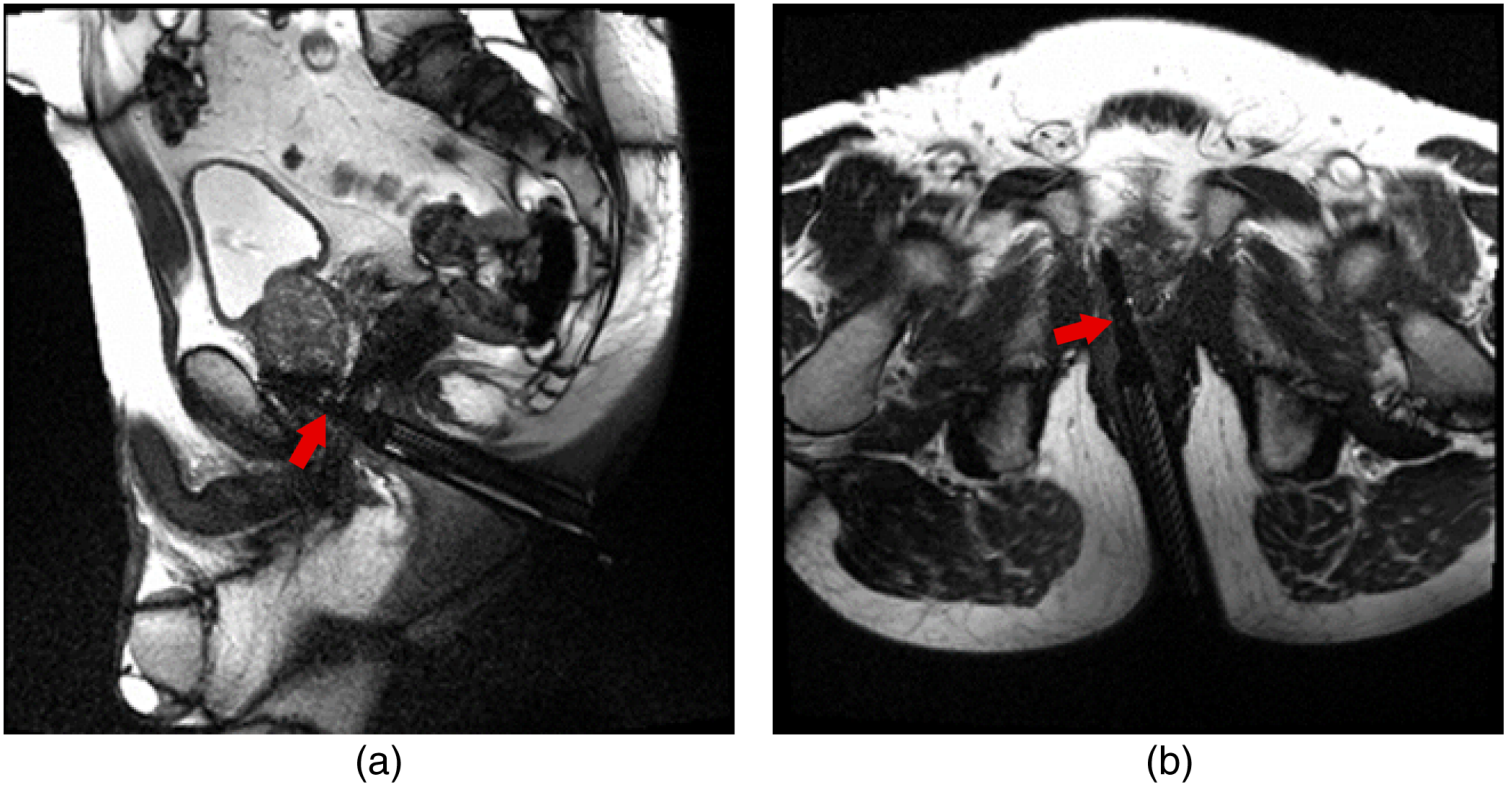
Examples of a sagittal (a) and transversal oblique (b) scan, with needle guides visible on both images. Notice the black artifact beyond the needle guide (red arrow) as the dataset includes confirmation scans with the MR-compatible needle in place, acquired immediately after a biopsy sampling.

Each multislice scan is aligned along a sagittal or a transversal oblique plane that aligns with the needle guide (Fig. 1). These scans focus on the needle guide, with the central slice most closely representing the lengthwise center of the structure. The needle guide has a length of 115 mm and a diameter of 11 mm, and given a slice thickness of 3 mm, on occasion, the needle guide might not always be visible in the first and fifth slices. The 3D multislice MRGB dataset is transformed into a simulated temporal plus 2D dataset by considering each slice as a moment in time. We thus treated each slice as a distinct timeframe to emulate a temporal dimension, effectively transforming the x−y−z space into an x−y−t space intended to emulate temporal MRI. This setup simulates the experience of tracking the passage of a needle through a slice.

### 2.2 Data Annotation

We extracted a subset of 1009 scans of 203 patients from the MRGB dataset for annotation to facilitate testing and image segmentation model training. These scans were extracted by aggregating all scans from a randomly selected patient repeatedly until the total number of scans exceeded 1000. The annotation process involved marking the central slice of each scan with a line from the back to the tip of the needle guide. This annotation work was split between two observers (Noordman CR, Borgers SJW, supervised by Fütterer JJ). The line’s endpoints defined the centers of a hypothetical cylindrical boundary. Any voxel touching or inside this boundary was included in the final annotation. A total of 1002 scans were annotated, excluding the remaining seven scans due to the visual absence of a needle guide. Within the annotated group, 508 and 494 scans aligned with the sagittal and transversal oblique planes.

### 2.3 Preprocessing

Undersampled k-space data are acquired synthetically, from DICOM images as obtained from the scanner, by performing a Fourier transform on the images. All images are normalized by scaling the 95th percentile intensity to 1, linearly scaling all other values to within [0, 1]. An undersampling mask is then applied, zeroing k-lines equal to the desired level of undersampling. The mask follows a Gaussian random sampling pattern with zero mean, zeroing fewer lines at the spatial low-frequency regions, described in further detail in Ref. 15. Rician noise was added to the synthetic k-space data, sampled using a mean of σ=0.05.

### 2.4 Algorithms

#### 2.4.1 Image reconstruction network

Earlier deep learning–based image reconstruction models for dynamic MRI employed a cascade of convolutional neural networks (CNNs), where the image reconstruction process is an iterative process with each sequential step independently parameterized.^16^ We adopted the convolutional recurrent neural network for MR image sequences (CRNN-MRI) model for iterative dynamic MR image reconstruction due to its relative simplicity and efficiency in reconstructing spatiotemporal data.^13^ This model can propagate information across iterations and time in a recurrent manner and is encapsulated in three convolutional recurrent units (CRNN), which evolve over iterations, and a bi-directional convolutional recurrent unit (BCRNN), which evolves over iterations and time. We modified the original implementation, found at github.com/js3611/Deep-MRI-Reconstruction, which minimized the pixel-wise mean square error to achieve accurate results across the entire image. Instead, we used the structural similarity index measure (SSIM), which encourages the network to optimize for structural similarity in both the anatomy and the instrument. Otherwise, we followed the hyperparameters as described in “Proposed-B” of Ref. 13: 10 iterations of 5 layers: a BCRNNN unit, three CRNN units and a CNN unit, using a kernel size of 3 and 128 filters. For more details, see Sec. S2 in the Supplementary Material.

#### 2.4.2 Instrument segmentation network

The needle guide instrument in the image reconstructions was segmented using a baseline nnU-Net, employed immediately following the output of the image reconstruction network.^17^ This U-Net–based implementation automatically configures all hyperparameters to optimally suit the dataset’s characteristics. The training was conducted using nnU-Net’s default trainer and planner algorithms, using the “2d” configuration.

#### 2.4.3 Principal component analysis for instrument position

The main objective of real-time interventional tracking tasks is to track the instrument tip. For each frame, precisely locating the tip reduces the chance of that instrument moving out of the field of view and other out-of-distribution errors. Principal component analysis was applied to determine the direction vector of the segmentation, closely following the method described in Ref. 18: the centroid of the segmentation is first computed, and the covariance matrix of the segmentation’s spatial coordinates is used to extract the principal eigenvector, which represents the instrument’s main axis. The final position vector (i.e., the instrument tip) is determined by selecting the endpoint of the longest line nearest to the image’s center.

The image reconstruction models were trained using the unannotated dataset and divided into a training and validation dataset using an 80/20 split. The image segmentation models were trained using the annotated dataset and divided into train, validation, and test sets using a 68/17/15 split. The annotated test dataset (n=1002×0.15=151) reports these results and comprises 75 sagittal scans and 76 transversal oblique scans. The image reconstruction dataset, annotated training and validation dataset, and annotated test dataset each comprised a distinct group of patients. (For more details on dataset splits, see Sec. S3 in the Supplementary Material.)

### 2.5 Experimental Analysis

We evaluated the quality of reconstruction and segmentation of our model with a reconstruction variant model restricted to using only spatial information (i.e., a nontemporal model) to determine the impact of exploiting temporal information on performance for this task by replacing the BCRNN module with the regular CRNN module. Reconstruction results were evaluated using SSIM, Dice score coefficient (DSC), instrument prediction success rate, and the ITP error. DSC measures the quality of the segmentation of the medical instrument present in the images by measuring the overlap between the segmentation of the needle guide in the predicted image and the annotation, where measurements range from 0 to 1, with DSC=1 suggesting perfect agreement with the annotation. The instrument prediction success rate evaluates the rate of successful predictions, specifically whether a model outputs any segmentation or whether a reader marks a scan as containing an instrument. The ITP error is defined as the millimeter distance between the (successfully) predicted and annotated positions of the needle guide tip in the center slice of a scan.

We trained models at increasing levels of undersampling to identify the maximum noninferior undersampling rate at which a model could accurately predict the ITP. According to a study on the clinical accuracy of an MRGB robotic prostate biopsy system, the maximum error should be less than 5 mm to avoid missing the targeted lesion, with the reported needle guide placement error having a mean of 2.5 mm and a standard deviation of 1.6 mm.^19^ Assuming the ITP error is a fair surrogate replacement for the needle guide placement errors, we considered reconstructions at a level of undersampling as noninferior if more than a pragmatically chosen 95% of ITP errors remained below this clinically relevant margin of 5 mm. If the 95% confidence interval (CI) proportion of ITP errors did not lie fully above this margin, that and subsequent undersampling rates were deemed inferior, inaccurate, and nonviable.

All experiments were conducted on a workstation equipped with an Intel Xeon Gold 6238R CPU (28 cores, 2.20 GHz), 64 GB of RAM, and an NVIDIA A100 GPU with 40 GB of VRAM.

### 2.6 Reader Study

We conducted a reader study that evaluated the accuracy of radiologists in locating the needle guide tip using the reconstructed output of our best-performing model. This task mirrors the segmentation model’s process. Our goal was to evaluate the difficulty of the problem as the level of undersampling increases and to provide a human reference standard for this task. Seven radiologists (three centers with a median 8 years of experience) took part, of which four readers have a history of practicing at the center where our dataset originates, and five readers have experience with prostate in-bore biopsies (median 9 years of prostate biopsy experience). Readers were instructed to identify the tip of the needle guide or to indicate that no needle guide could be interpreted.

Readers were given 15 sagittal and 15 transversal oblique randomly selected fully sampled scans from our 151-count annotated test dataset split. Following these, they were given these same scans, but as undersampled reconstructions as output by our models, in random order. Due to expected inter-reader variation in determining the needle guide tip, readers’ answers to the fully sampled scans have been defined as their personal reference standard, as ITP error assumes error caused by the poor image quality of the undersampled reconstructions and not error due to disagreement with the annotated dataset. For more details on the reader study reading workflow, see Sec. S4.1 in the Supplementary Material.

### 2.7 Statistical Analysis

To assess noninferiority, we computed Wilson score confidence intervals for the proportion of ITP errors below 5 mm. A one-sided test at 95% confidence is used to assess whether the model’s performance was noninferior to segmenting fully sampled (1×) images. A level of undersampling was deemed noninferior if the lower bound of the 95% CI for this proportion was ≥95%. If the lower bound dropped below this threshold, that and subsequent undersampling rates were considered nonviable. For all continuous variables, we report the mean, and 95% CI Student’s paired t-test with Bonferroni adjustment was used to account for multiple comparisons. All P-values are two-sided, with statistical significance set at P<0.05. Analysis was conducted using Python (python-v3.12, scipy-v1.14).

## 3 Results

**Fig. 2:**
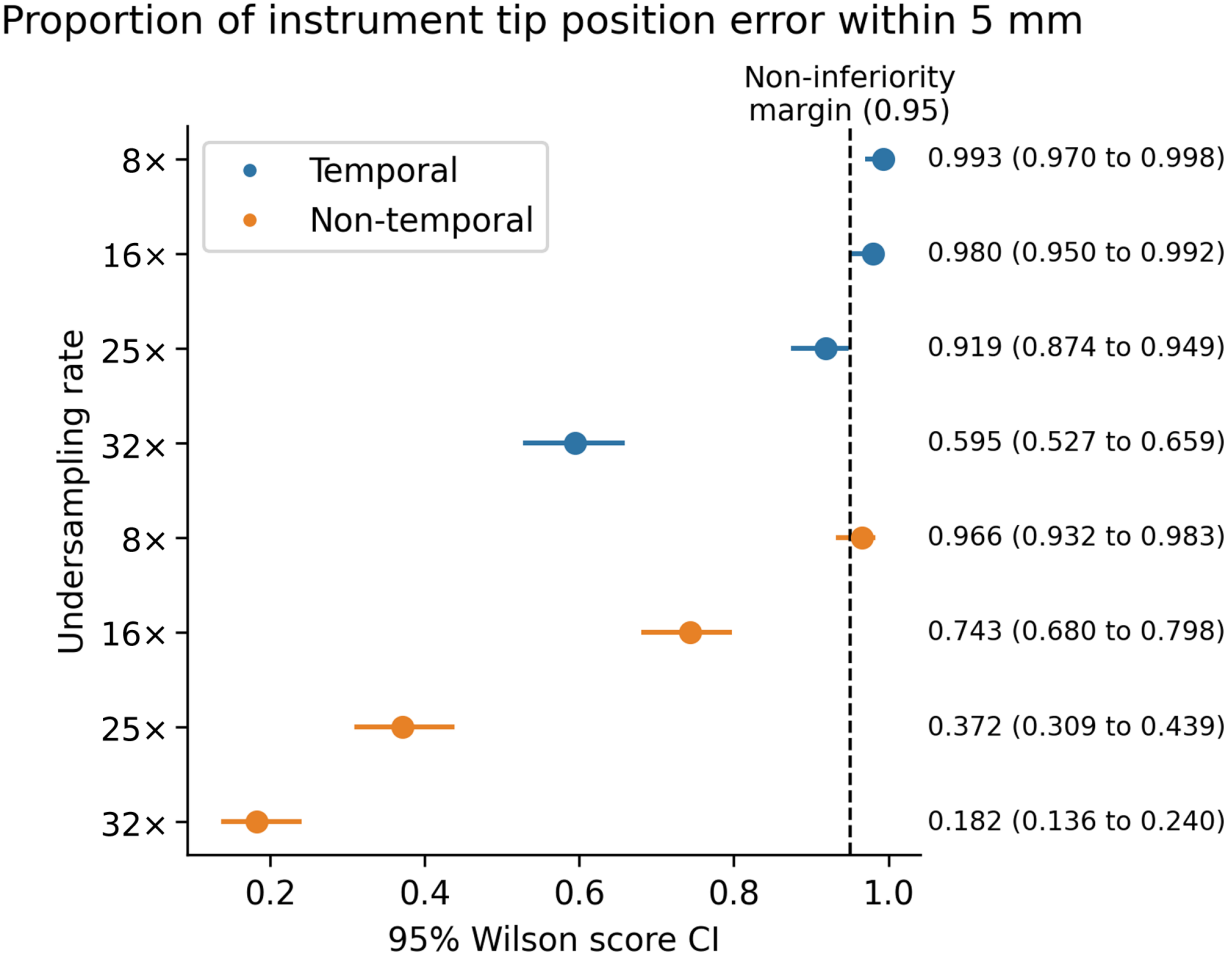
One-tailed Wilson score confidence intervals at 95% confidence, used to assess noninferiority compared with the reference standard.

Figure 2 presents the primary outcome of our study, demonstrating that noninferiority was achieved only for the 8× and 16× undersampling rates when using the temporal model. At undersampling rates beyond 16×, reconstructions were inferior to those from our reference standard: fully sampled images. The nontemporal model failed to show any noninferiority across all tested undersampling rates, with an 8× undersampling rate yielding an inconclusive result.

**Fig. 3:**
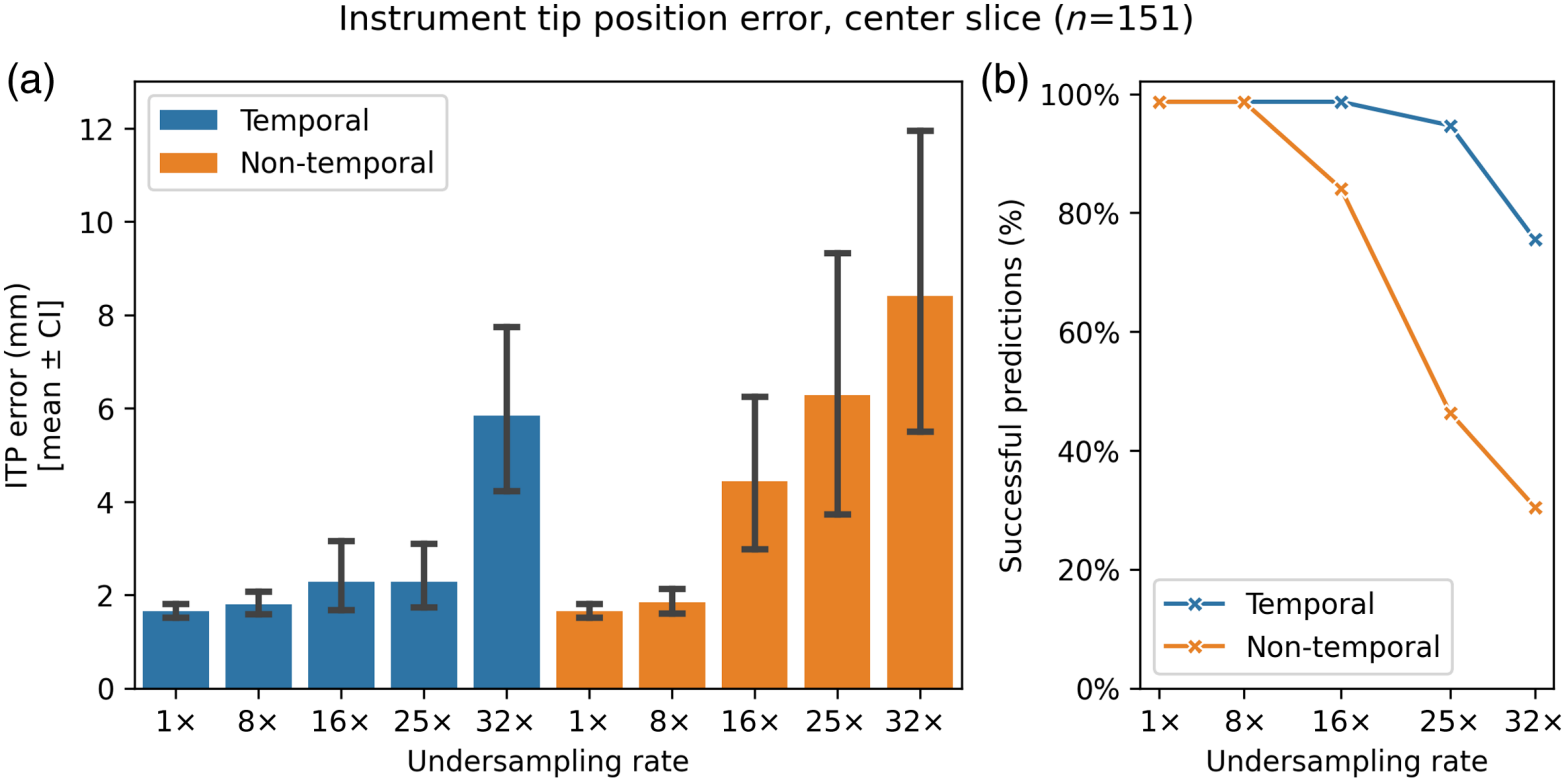
(a) Evaluation of the instrument tip position (ITP) error, calculated from the annotated tip position, for the central slice using the temporal and nontemporal models’ reconstruction outputs (n=151). Values are presented as mean±95% CI. (b) The percentage of scans with successful predictions; unsuccessful predictions are due to model segmentation failures.

Figure 3 presents the ITP error for the central slices of the test dataset, comparing temporal and nontemporal reconstruction models across various undersampling rates (1×, 8×, 16×, 25×, and 32×). Figure 3(a) shows the mean error with error bars representing the 95% CI, and Fig. 3(b) shows the instrument prediction success rates. Only successful predictions are included to maintain a fair comparison between models, as failed predictions provide no ITP value or error value. Table 1 summarizes the means, 95% CIs, mean differences in mm, and P-values for the ITP error compared with the reference standard ITP error across both models and undersampling rates.

**Table 1:** Comparisons of instrument tip prediction (ITP) error, calculated from the annotated tip position, between the reference standard and undersampled reconstruction output prediction using the test set.

	R	ITP error, mm, n=151	Mean difference, mm	P-value
	1×	1.65 (1.50 to 1.81)	—	—
Temporal model	8×	1.80 (1.59 to 2.08)	0.15	0.130
	16×	2.28 (1.68 to 3.31)	0.63	0.090
	25×	2.29 (1.73 to 3.15)	0.64	0.104
	32×	5.84 (4.28 to 7.71)	4.19	<0.001
Nontemporal model	8×	1.85 (1.59 to 2.17)	0.20	0.115
	16×	4.43 (2.92 to 6.24)	2.78	0.001
	25×	6.27 (3.90 to 9.07)	4.62	0.001
	32×	8.40 (5.57 to 12.19)	6.75	<0.001

Data in parentheses are 95% CIs. P-values are calculated using Student’s paired t-test and are statistically significant at the Bonferroni-adjusted threshold of P<0.0125. ITP, instrument tip prediction; R, undersampling rate.

**Fig. 4:**
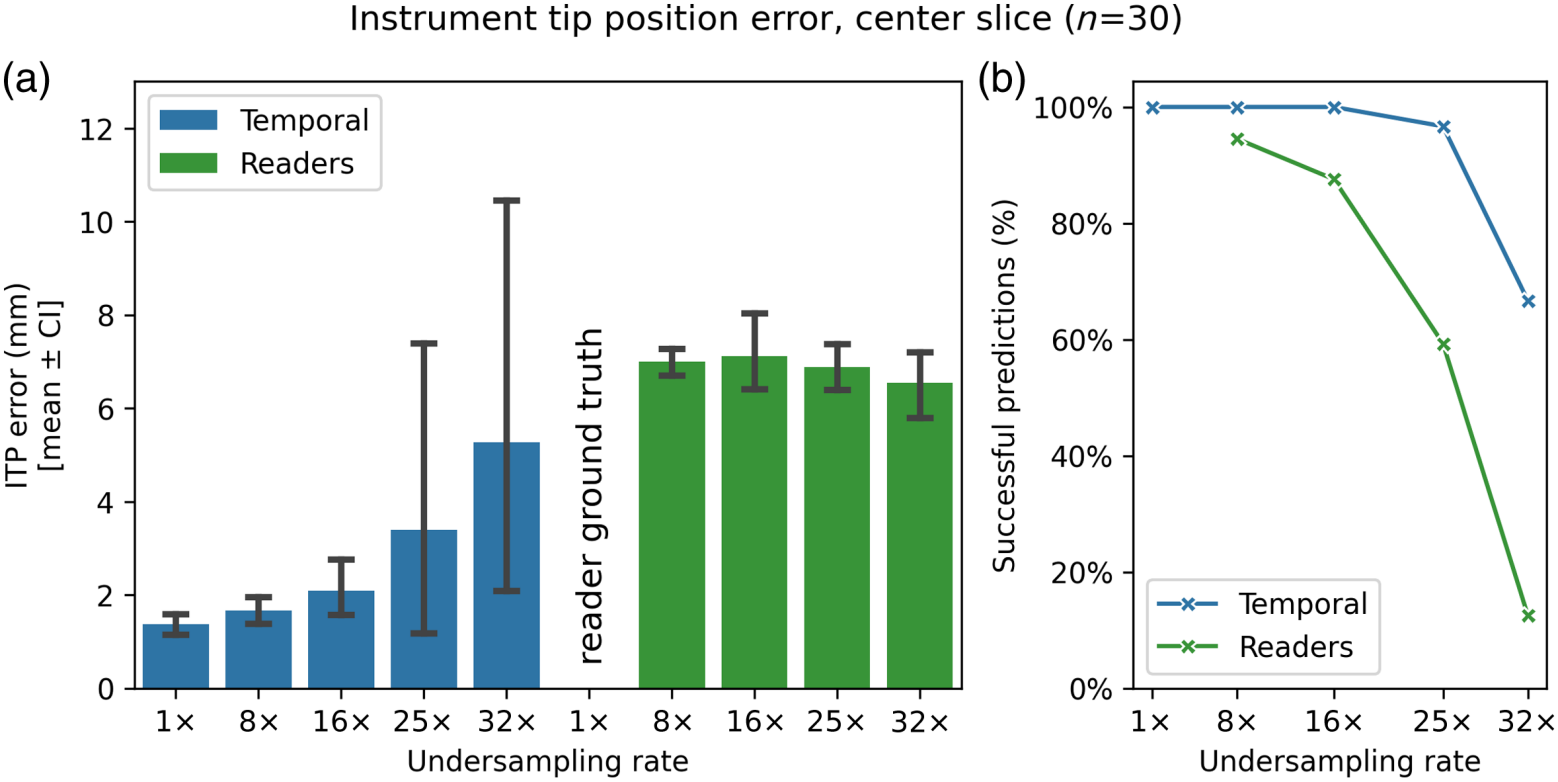
(a) Evaluation of the instrument tip position (ITP) error, calculated from the annotated tip position, for the central slice using the temporal reconstruction and segmentation outputs compared with reader evaluations. Values are presented as mean±95% CI. ITP errors for readers were based on their answers to the 1× images. (b) The percentage of scans with successful predictions; unsuccessful predictions are due to model segmentation failures or reader judgments that the instrument tip was not visible.

Figure 4 presents a comparison similar to Fig. 3 but evaluates the ITP error of the temporal model versus combined reader evaluations using the reader study test subset. Readers and segmentation models utilized the temporal model’s reconstruction output to predict ITPs. As with the previous figure, only successful predictions are included. The pipeline demonstrates superior accuracy over human readers in predicting ITPs, with P≤0.001 for all undersampling rates above 1×. Although the ITP error for readers remained stable across undersampling rates with a mean of 6.88 mm ([7.00+7.11+6.87+6.55]/4), the instrument prediction success rate declined rapidly from 28 of 30 (93%) scans at an 8× undersampling rate to 18 of 30 (60%) scans at a 25× undersampling rate. Table 2 details the means, 95% CIs, and P-values, highlighting the significant differences in ITP error between the temporal model and human readers. For the individual reader performances, see Sec. S4.2 in the Supplementary Material.

**Table 2:** Performance comparisons of instrument tip prediction (ITP) errors, calculated from the annotated tip position; model compared with the combined assessment of seven readers using the reader study test subset.

	R	Temporal model, n=30	Seven readers, n=30	P-value
ITP error, mm	1×	1.37 (1.15 to 1.60)	—	—
	8×	1.67 (1.38 to 1.92)	7.00 (6.69 to 7.29)	<0.001
	16×	2.09 (1.59 to 2.72)	7.11 (6.48 to 8.12)	<0.001
	25×	3.40 (1.20 to 7.39)	6.87 (6.38 to 7.40)	<0.001
	32×	5.27 (2.08 to 10.60)	6.55 (5.77 to 7.24)	0.001

Data in parentheses are 95% CIs. P-values are calculated using Student’s paired t-test and are statistically significant at the Bonferroni-adjusted threshold of P<0.0125. ITP, instrument tip prediction; R, undersampling rate.

**Fig. 5:**
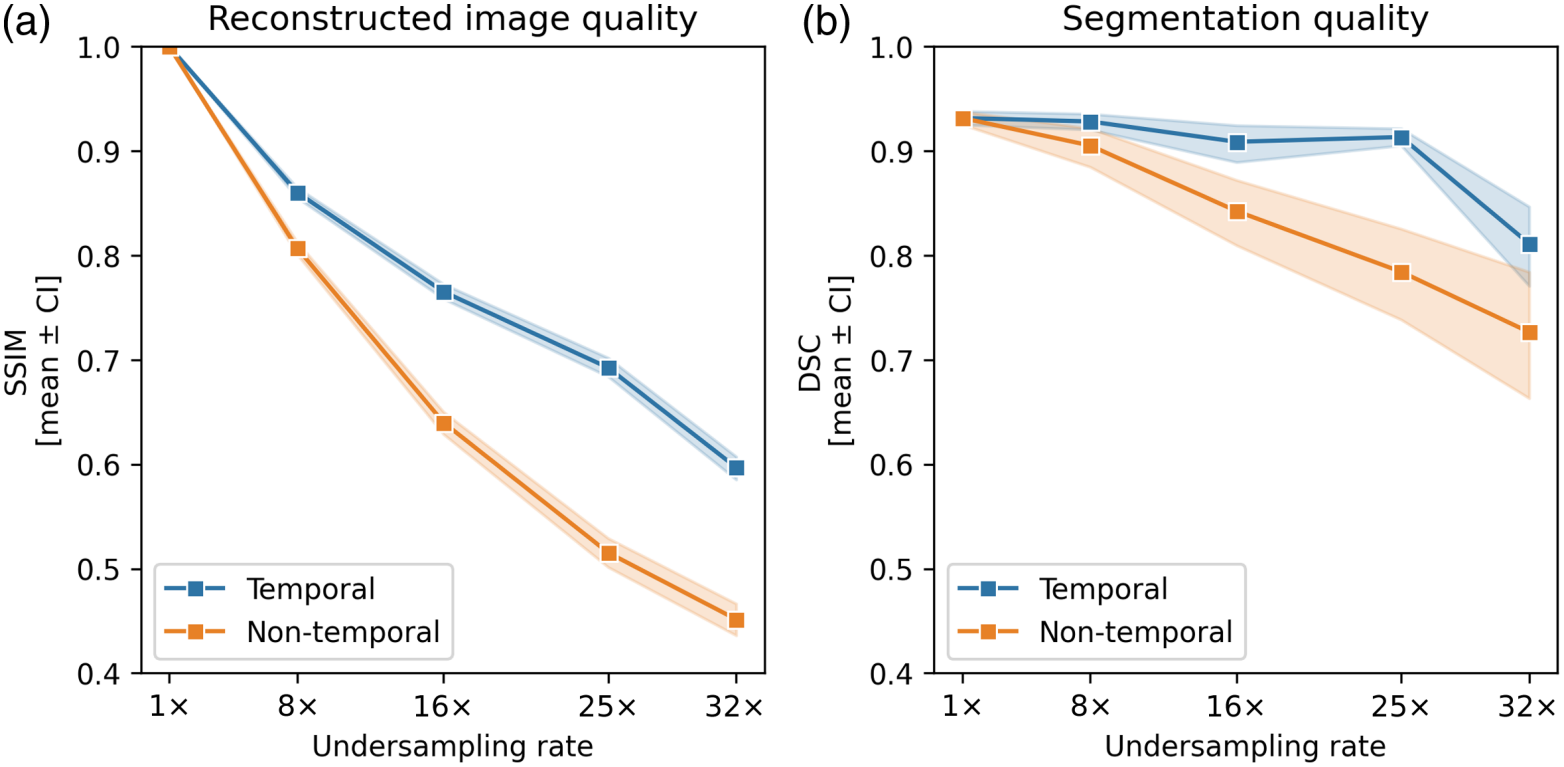
Evaluation of the (a) reconstruction and (b) segmentation quality of the central slice (n=151). Values are presented as mean±95% CI. The plots compare the efficacy of a temporal model against a nontemporal model with rising undersampling levels, assessing structural similarity index measure (SSIM) or Dice score coefficient (DSC). Note that the DSC plot only includes samples containing a segmentation (i.e., the segmentation model could identify the instrument in the image).

Figure 5 evaluates the performance of temporal and nontemporal models in reconstructing and segmenting the test dataset, examining the SSIM and DSC of the center slice, with shaded areas showing a 95% CI. Instances where the segmentation model failed to produce any output are excluded from the DSC evaluation. For SSIM and DSC, for all tested undersampling rates above 1, the temporal model shows significant improvement compared with the nontemporal model (all SSIM comparisons P<0.001, all DSC comparisons P<0.01). Table 3 shows mean, 95% CIs, and P-values for each undersampling rate for both temporal and nontemporal models of the SSIM, DSC, and ITP error metrics.

**Table 3:** Performance comparisons of reconstruction, segmentation, and instrument tip prediction error; temporal model compared with the nontemporal model using the test set.

	R	Temporal model, n=151	Nontemporal model, n=151	P-value
SSIM	1×	1.00 (1.00 to 1.00)	1.00 (1.00 to 1.00)	—
	8×	0.86 (0.86 to 0.87)	0.81 (0.80 to 0.81)	<0.001
	16×	0.77 (0.76 to 0.77)	0.64 (0.63 to 0.65)	<0.001
	25×	0.69 (0.68 to 0.70)	0.52 (0.50 to 0.53)	<0.001
	32×	0.60 (0.59 to 0.61)	0.45 (0.44 to 0.47)	<0.001
DSC	1×	0.93 (0.93 to 0.94)	0.93 (0.92 to 0.94)	—
	8×	0.93 (0.92 to 0.94)	0.91 (0.89 to 0.92)	0.007
	16×	0.91 (0.89 to 0.92)	0.84 (0.81 to 0.87)	<0.001
	25×	0.91 (0.90 to 0.92)	0.78 (0.74–0.83)	<0.001
	32×	0.81 (0.77 to 0.85)	0.73 (0.66 to 0.78)	0.002
ITP error, mm	1×	1.65 (1.50 to 1.81)	1.65 (1.51 to 1.79)	—
	8×	1.80 (1.59 to 2.08)	1.85 (1.59 to 2.17)	0.773
	16×	2.28 (1.68 to 3.31)	4.43 (2.92 to 6.24)	0.023
	25×	2.29 (1.73 to 3.15)	6.27 (3.90 to 9.07)	0.002
	32×	5.84 (4.28 to 7.71)	8.40 (5.57 to 12.19)	0.017

Data in parentheses are 95% CIs. P-values are calculated using Student’s paired t-test and are statistically significant at the Bonferroni-adjusted threshold of P<0.0125. SSIM, structural similarity index measure; DSC, Dice’s similarity coefficient; ITP, instrument tip prediction; R, undersampling rate.

**Fig. 6:**
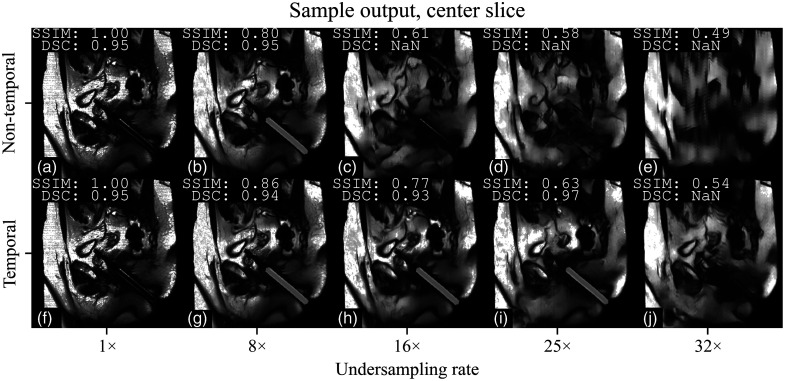
Visualization examples of a sagittal scan from the test set, showing the output reconstructions overlaid with the segmentations for both nontemporal (a)–(e) and temporal (f)–(j) models, across increasing levels of undersampling. Each image is annotated with its respective structural similarity index measure (SSIM) and Dice score coefficient (DSC) values. If the DSC is not “NaN,” its respective segmentation is also shown (with the exception of the first column).

Figure 6 offers a visual comparison of the reconstructions and segmentation output of both models. The nontemporal model shows noticeable echoing artifacts at higher undersampling rates. Smoothing artifacts are visible for both models but do not appear severe enough to obscure the needle guide entirely for the segmentation model.

## 4 Discussion

In this work, we leveraged one of the largest datasets to date for training deep learning models tailored to an iMRI task. We hypothesized that the model’s ability to use information over time would significantly enhance the quality of reconstructed images and improve the accuracy of ITP prediction. Our findings confirm this hypothesis, with the maximum noninferior undersampling rate being 16× for the temporal model and the nontemporal model, where spatiotemporal exploitation is inhibited, is incapable of performing image reconstruction that is noninferior to the reference standard.

We also demonstrated a significant improvement in SSIM across all undersampling rates, consistent with the findings in Ref. 13, and observed similar improvements in DSC. The SSIM and DSC comparison provides an insightful perspective on the model’s efficacy, revealing a nuanced interplay between image quality and segmentation accuracy. The weak correlation between SSIM, an indicator of overall image quality, and ITP error, our metric for evaluating task-specific performance, suggests that SSIM may not be reliable for gauging performance in specialized instrument tracking applications. As an illustrative example, Fig. 6(d) has a similar SSIM to Fig. 6(i) but failed to produce a segmentation output. The benefit of spatiotemporal exploitation is most evident at its maximum noninferior rate of 16×. At this rate, neither the readers nor the nontemporal model can reliably predict the instrument, achieving a success rate of 68% with a mean error of 7.11 mm and 84% with a mean error of 4.43 mm, respectively. By contrast, the temporal model predicts successfully 95% of the time, with a mean error of 2.28 mm.

To the best of our knowledge, this is the first report of a reconstruction and segmentation model for MRGB needle guide localization using undersampled input. Earlier research focused on segmenting percutaneous needles from fully sampled acquisitions,^2^^,^^20^^,^^21^ whereas the most comparable study reported a 1.28 mm mean needle tip localization error from segmentations of fully sampled MRGB scans.^22^

This study had limitations. The retrospective data were used to simulate a one-dimensional orthogonal movement in a 2D-time series, limiting generalization to any motion. Nonetheless, our clinicians perceived the setup as realistic and considered it representative of clinical practice. Second, we simulated k-space from imaging data, and although this is a common approach in various MRI reconstruction studies, we realize this may further limit generalizability. Finally, although Cartesian sampling offers a certain robustness against artifacts, it is not necessarily the most strategic choice for real-time guidance, where high temporal resolution is critical. Alternative sampling trajectories, such as radial or keyhole imaging, could be more advantageous as they naturally emphasize the k-space center and allow for higher acceleration.^23^^,^^24^ Radial trajectories may be particularly well suited for instrument tracking as the instrument is typically a long, thin structure. Future studies should explore these alternative trajectories to further optimize image reconstruction for interventional applications. However, in our future work, we plan to employ prospective intraprocedural pre-biopsy real-time k-space acquisitions, which will more accurately reflect real-world conditions.

This study demonstrated that spatiotemporal exploitation in MR image reconstruction may positively impact time-sensitive interventional tasks such as instrument tracking. Our approach has the potential to enhance clinicians’ capabilities by facilitating real-time instrument positioning, leading to more efficient and accurate MR-guided interventional radiology procedures.

## Supplementary Material

10.1117/1.JMI.12.3.035001.s01

## Data Availability

The data that support the findings of this study are available upon reasonable request.
