# Supplementary material for: Deep learning–based temporal MR image reconstruction for accelerated interventional imaging during in-bore biopsies
Source: J Med Imaging (Bellingham). 2025 Jun 3;12(3):035001. doi: 10.1117/1.JMI.12.3.035001 (PMC12131189; doi:10.1117/1.JMI.12.3.035001)
Supplement: Supplementary file 1 [file JMI_012_035001_SD001.pdf]

Supplemental Materials

S1 Patient Population

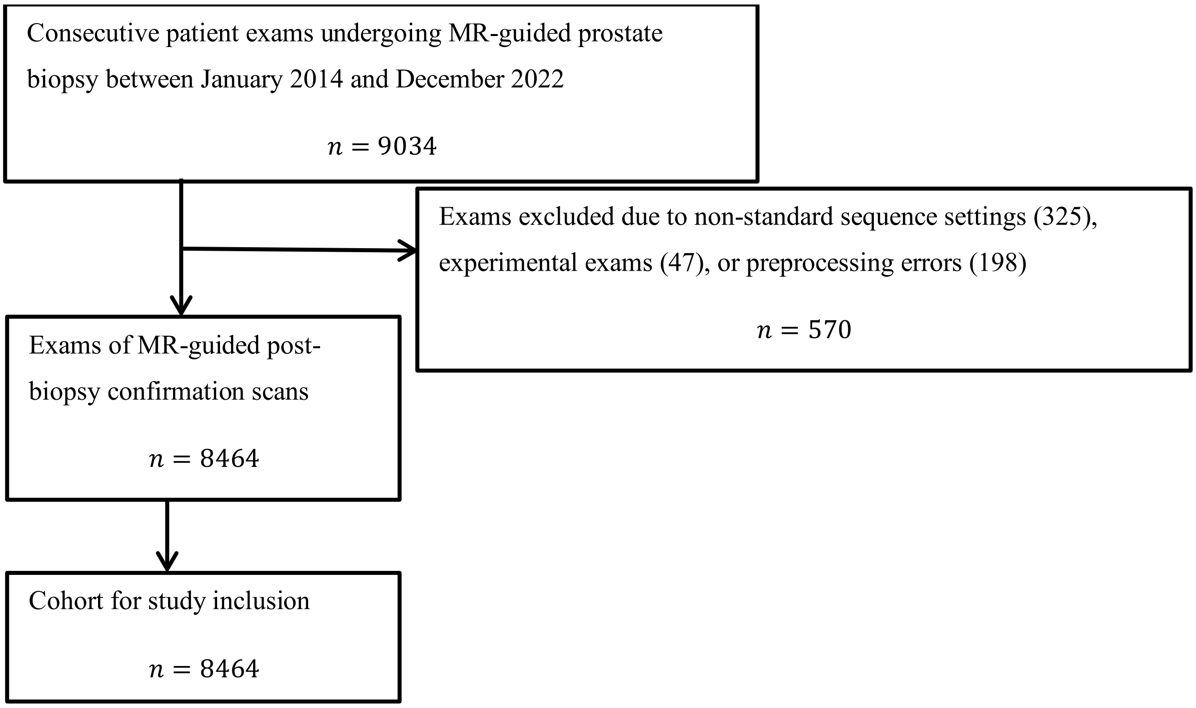

Fig. S1: Patient cohort selection flowchart.

S2 Model Development

Earlier dynamic MR deep learning-based image reconstruction models employed a cascade of convolutional neural networks (CNN), where the image reconstruction process is an iterative process with each sequential step independently parameterized [Ref. 16]. We adopted the CRNN-MRI model for iterative dynamic MR image reconstruction due to its relative simplicity and efficiency in reconstructing spatiotemporal data [Ref. 13]. This model can propagate information across iterations and time in a recurrent manner,

$$x_{rec} = f_N\left(f_{N-1}\left(\cdots\left(f_1(x_u)\right)\right)\right) \quad (1)$$

where x_{rec} is the network prediction, x_u is the undersampled k-space input, and f_n is the network function at the nth iteration. This general model is encapsulated in three convolutional recurrent units (CRNN), which evolve over iterations, and a bi-directional convolutional recurrent unit (BCRNN), which evolves over iterations and time.

These recurrent units are highly efficient as information gathered from previous iterations is propagated, effectively increasing the receptive field of each future iteration. The BCRNN comprises three convolutional layers: the previous iteration layer, the past time frame, and the future time frame. The bi-directional aspect is crucial, as it involves reconstructing the initial time frame using subsequent time frames and reciprocally reconstructing the last time frame using earlier time frames. These units are followed by a regular CNN, which projects the information back to image space, and a data consistency layer to keep the sampled k-space data consistent with the prediction [Ref. 16].

Using a simulated temporal dataset $\mathcal{D}$ with undersampled data as input and the fully sampled data as the target, a model is trained end-to-end by minimizing the structural similarity index measure (SSIM) between reconstructed data and the reference standard y:

$$\mathcal{L}(\theta) = \frac{1}{N_{\mathcal{D}}} \sum_{(x^0, y) \in \mathcal{D}} \left\| SSIM(y, x^i) \right\| \quad (2)$$

where x^i denotes the predicted image at the final iteration of the network, θ is the set of network parameters, and $N_{\mathcal{D}}$ is the number of training samples. The original implementation minimized the pixel-wise mean square error, encouraging accurate results across the entire image. SSIM appeared more suitable, as it may encourage the network to optimize for structural similarity in anatomy and the instrument instead.

For all (B)CRNN units, we used the Proposed-B parameter settings from Ref. 13. Gradient clipping is applied with a maximum norm of 1 to prevent gradient explosions during training. The network weights are initialized using He initialization [Ref. 25]. We employed the Adam optimizer with an exponential learning rate, initialized to 1^{-3} with a gamma decay rate of 0.95, and trained each model for 50 000 backpropagations. The nnU-Net segmentation model has a predefined data preprocessing and augmentation pipeline, detailed in Ref. 17, and is trained for 250 000 backpropagations. All computations were performed on a NVIDIA DGX A100 using one SXM4 40Gb GPU and an Intel Xeon Gold 6238R processor with 32 GB RAM.

S3 Dataset characteristics

Table S1 Dataset splits across deep learning model training, validation and testing

	**Train**[1]	**Validation**[1]	**Test**[2]	**All**
Number of patients				
CRNN-MRI	100% (1086/1086)			84% (1086/1289)
nnU-Net	86% (175/203)		14% (28/203)	16% (203/1289)
Number of scans				
CRNN-MRI				
Sagittal	75% (2992/3739)	25% (747/3739)		50% (3739/7455)
Oblique	75% (2973/3716)	25% (743/3716)		50% (3716/7455)
nnU-Net				
Sagittal	68% (346/508)	17% (87/508)	15% (75/508)	51% (508/1002)
Oblique	68% (334/494)	17% (84/494)	15% (76/494)	49% (494/1002)

[1] Training and validation sets did not split scans between patients.

[2] CRNN-MRI has no test set, as evaluations are only performed using the nnU-Net test set.

S4 Reader Study

S4.1 Reading Workflow

Readers were provided with 15 sagittal and 15 oblique fully sampled cases and were instructed to draw a single line from the bottom to the tip of the needle guide (Figure S2). Subsequently, readers were presented with these same 30 cases in a randomized order but undersampled and reconstructed by our temporal model at undersampling rates 8, 16, 25, and 32. If readers could not accurately interpret a needle guide with certainty, they were instructed to indicate the needle guide was not visible. Their answer to the first 30 (fully sampled) cases is defined as their reference standard, as instrument tip prediction (ITP) error assumes error caused by poor image quality of the undersampled reconstructions and not error due to disagreement with the annotated dataset. Figures S3-S8 show individual reader performance and are compared to the temporal model for easier contextualizing these results.

The reader study was handled through the https://grand-challenge.org/ platform, using the Cirrus Core viewer (v2024.05.2).

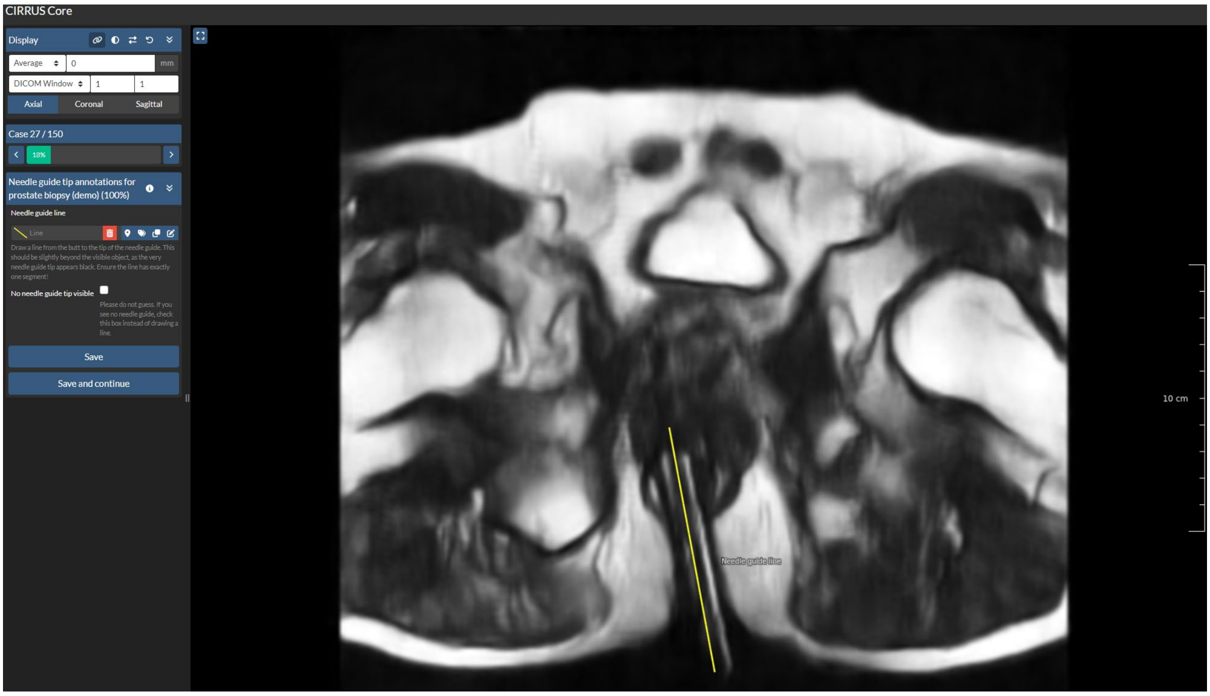

Fig. S2 Reader interface used during the reader study.

S4.2 Individual Reader Performance

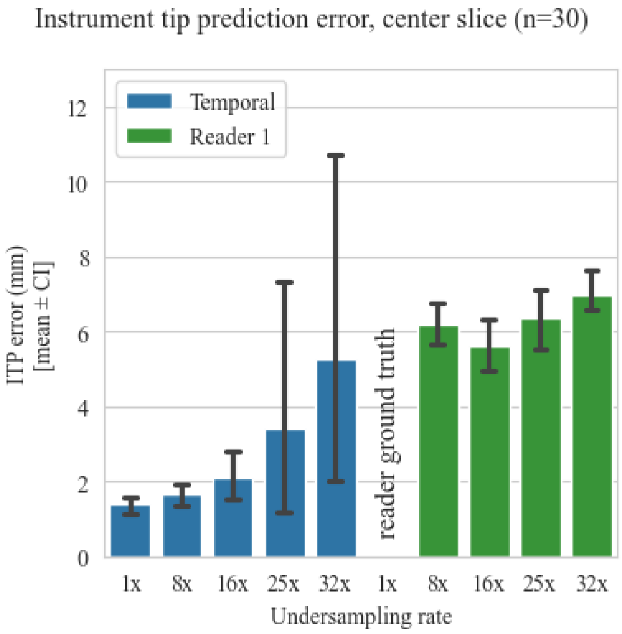

Fig. S3 Temporal model versus Reader 1

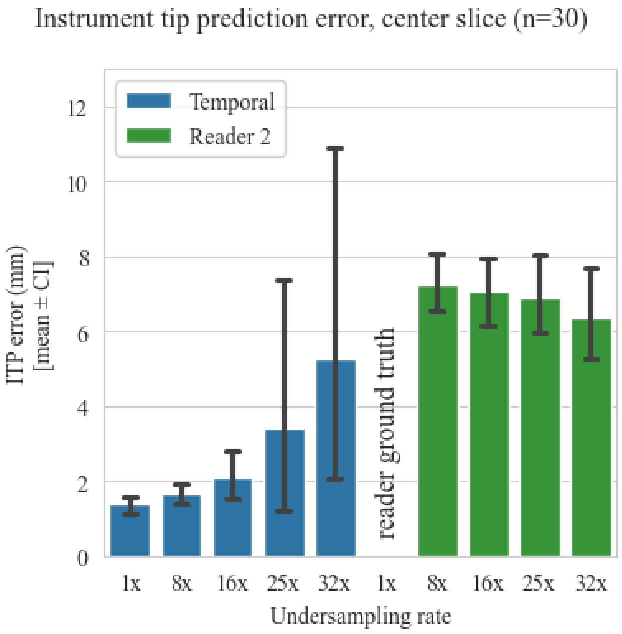

Fig. S4 Temporal model versus Reader 2

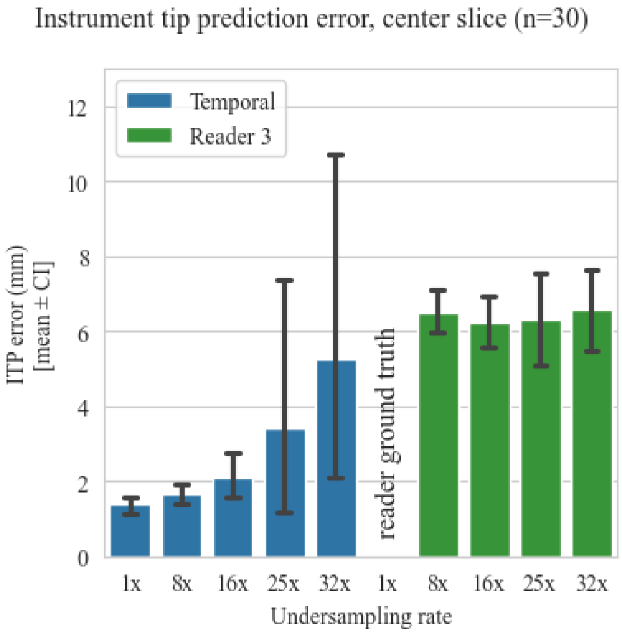

Fig. S5 Temporal model versus Reader 3

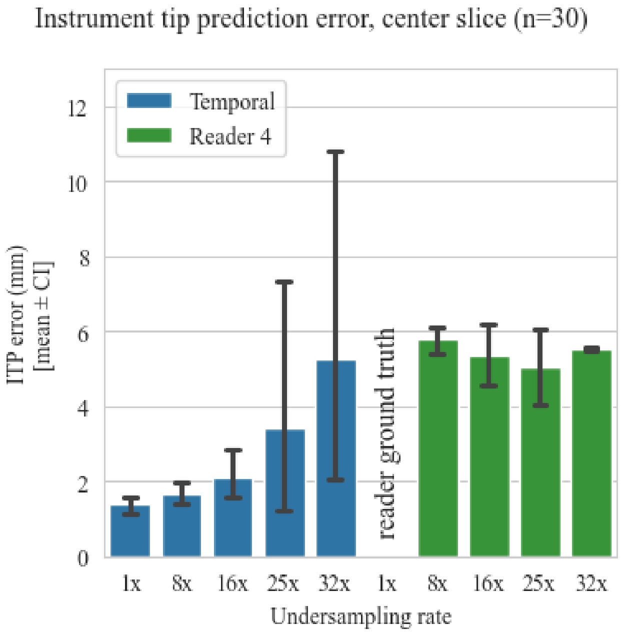

Fig. S6 Temporal model versus Reader 4

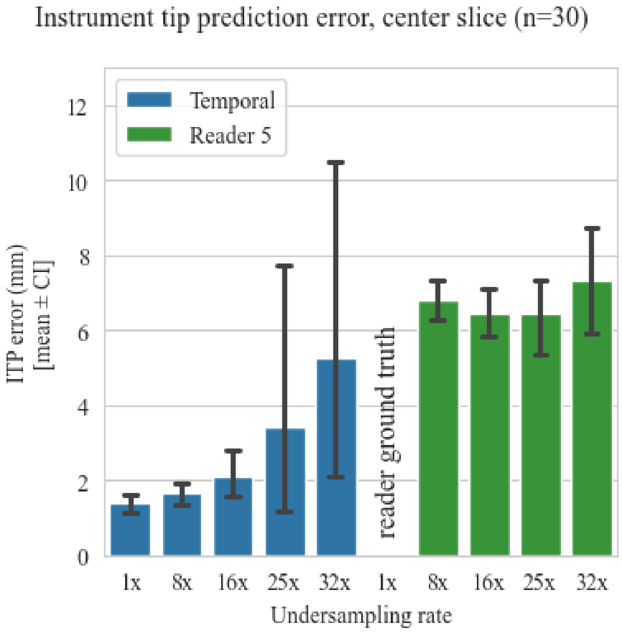

Fig. S7 Temporal model versus Reader 5

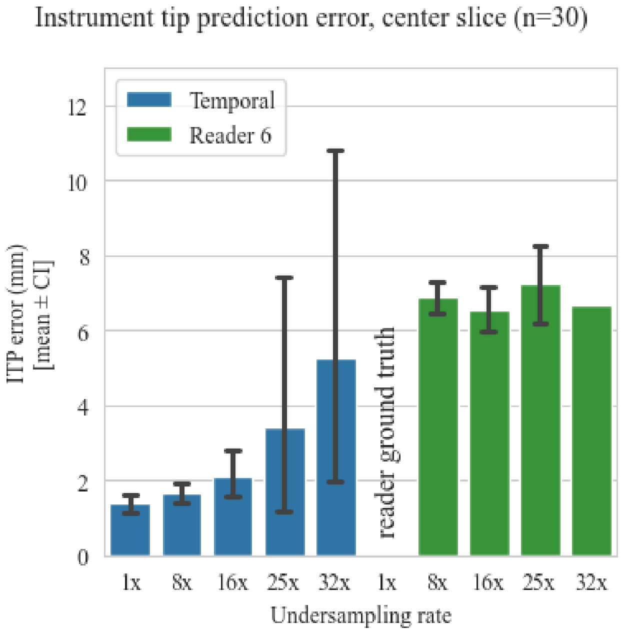

Fig. S8 Temporal model versus Reader 6

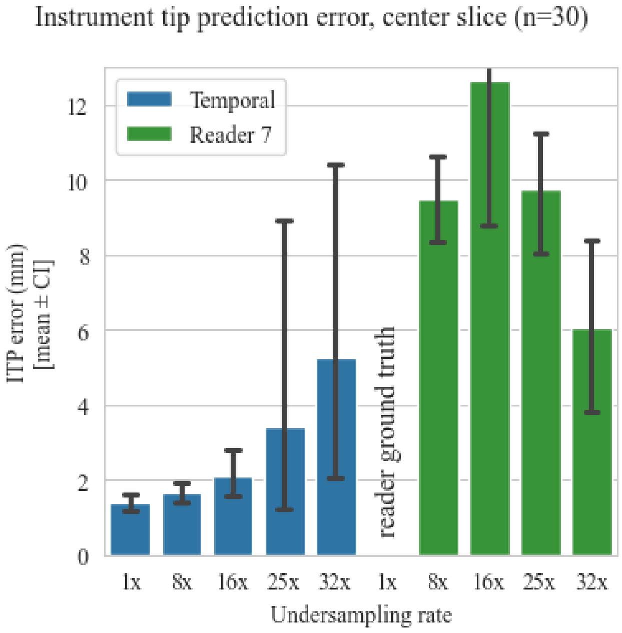

Fig. S9 Temporal model versus Reader 7
